# Clinical Translation of Tethered Confocal Microscopy Capsule for Unsedated Diagnosis of Eosinophilic Esophagitis

**DOI:** 10.1038/s41598-018-20668-8

**Published:** 2018-02-08

**Authors:** Nima Tabatabaei, DongKyun Kang, Minkyu Kim, Tao Wu, Catriona N. Grant, Mireille Rosenberg, Norman S. Nishioka, Paul E. Hesterberg, John Garber, Qian Yuan, Aubrey J. Katz, Guillermo J. Tearney

**Affiliations:** 1 Department of Mechanical Engineering, Lassonde School of Engineering, York University, Toronto, ON M3J 1P3 Canada; 2 Wellman Center for Photomedicine, Massachusetts General Hospital Harvard Medical School, Boston, MA 02114 USA; 3 Department of Medicine, Massachusetts General Hospital Harvard Medical School, Boston, MA 02114 USA; 4 Department of Pathology, Massachusetts General Hospital Harvard Medical School, Boston, MA 02114 USA; 5 Harvard-MIT Division of Health Science and Technology, Cambridge, MA 02139 USA

## Abstract

Esophagogastroduodenoscopy (EGD) is a widely used procedure, posing significant financial burden on both healthcare systems and patients. Moreover, EGD is time consuming, sometimes difficult to tolerate, and suffers from an imperfect diagnostic yield as the limited number of collected biopsies does not represent the whole organ. In this paper, we report on technological and clinical feasibility of a swallowable tethered endomicroscopy capsule, which is administered without sedation, to image large regions of esophageal and gastric mucosa at the cellular level. To demonstrate imaging capabilities, we conducted a human pilot study (n = 17) on Eosinophilic Esophagitis (EoE) patients and healthy volunteers from which representative cases are presented and discussed. Results indicate that, compared to endoscopic biopsy, unsedated tethered capsule endomicroscopy obtains orders of magnitude more cellular information while successfully resolving characteristic tissue microscopic features such as stratified squamous epithelium, lamina propria papillae, intraepithelial eosinophils, and gastric cardia and body/fundic mucosa epithelia. Based on the major import of whole organ, cellular-level microscopy to obviate sampling error and the clear cost and convenience advantages of unsedated procedure, we believe that this tool has the potential to become a simpler and more effective device for diagnosing and monitoring the therapeutic response of EoE and other esophageal diseases.

## Introduction

Esophagogastroduodenoscopy (EGD) is a widely used procedure for the diagnosis, screening and treatment of esophageal, gastric, and small-bowel disorders. In United States alone, 6.9 million EGD procedures were performed in 2009 with an estimated cost of $12.3 billion dollars^1^. Aside from the financial burden, EGD procedures are time consuming and inconvenient for patients who, because of sedation, need to take an entire day off from work. Moreover, since endoscopic biopsy only samples a small fraction of the organ, it is often subject to sampling error, resulting in the need to take many biopsies and sometimes leading to false negative diagnoses. The situation is particularly challenging for patients with certain allergic disorders where many sedated EGDs with biopsies may be required for the diagnosis and the management of the disease.

A case in point is the recently recognized allergic/immune condition referred to as Eosinophilic Esophagitis (EoE)^2^. EoE is an inflammatory condition of the esophagus that occurs in response to certain foods or allergens. At the microscopic level, EoE is manifested by eosinophilic infiltration within the esophageal wall causing debilitating symptoms such as dysphagia, nausea, and vomiting^3,4^. When untreated, long term esophageal eosinophilic inflammation can precipitate stricture formation that impairs esophageal function and leads to food impaction^4^. Approximately 300,000 people in the US have EoE^3,5^ and the incidence of newly diagnosed EoE is estimated at 30,000/year^3,5^. Because of the impact of EoE on quality of life, as well as the concern that this disease may progress to esophageal fibrosis with unknown long-term risks, experts recommend that EoE be treated until symptoms and the eosinophilic infiltrate are resolved^4,6,7^. The two main treatments of EoE are topical corticosteroids or dietary therapy. While steroids are helpful for placating symptoms and histologic normalization of the esophagus, symptoms and eosinophils frequently recur when the steroids are stopped^8^. The definitive therapy is removal of the offending allergen from the diet. To determine the culprit, patients are placed on restricted or elemental diets, a food antigen group is introduced, and EGD/biopsy is performed to determine if the eosinophilia recurs^9^. This iterative diagnosis of the culprit antigen in EoE heavily relies on repeat sedated EGDs with a large number of biopsies^10^. Consequently, EoE patients are subject to numerous expensive EGDs which are not only time consuming but also difficult to tolerate.

The compelling need for a less invasive and more cost-effective means of comprehensive microscopic interrogation of the GI tract (e.g., identifying eosinophils in EoE patients) has triggered the invention and development of several new technologies that could be less invasive and more specific for EoE. Confocal laser endomicroscopy (CLE) is one such technology that performs confocal microscopy using fluorescent dyes, applied either systemically or topically, to visualize cellular features^11^. The most common application of CLE in the esophagus is the interrogation of suspicious lesions in patients presenting with Barrett’s esophagus (BE)^12^. Nevertheless, in 2011, Neumann *et al*. reported a single case of an 18-year-old male subject presenting with symptoms and EGD findings consistent with acute EoE (e.g., with some narrow, long, linear channels)^13^. While CLE observations suggested microstructural hallmarks of EoE, such as dilated intercellular spaces, fluorescein leakage, and small cells within the intercellular spaces suspicious for eosinophils, to our knowledge, CLE is not currently used for diagnosis or screening of EoE patients. Multi-photon Fluorescence microendoscopy (MPM)^14^ and second harmonic generation scanning endomicroscopy^15^ are alternatives to CLE that provide high-resolution morphological and spectroscopic information on tissue states without the need for labeling. In a study reported by Safdarian *et.al*.^14^, EGD biopsy samples from EoE subjects were imaged by MPM and eosinophil counts and distributions were found and compared to that from histology. Despite promising results, both CLE and multi-photon technologies, in their present forms, would require sedated endoscopy for their utilization in patients. They also have relatively small tissue area coverage and thus, similar to endoscopic biopsy, are subject to sampling error. Other less invasive and more global sampling tests such as a string test^16^ and tethered capsule sponge^17^ have been proposed for the diagnosis of EoE. While early pilot results suggest diagnostic capacity, these tests cannot localize eosinophilic infiltration and cannot specifically count eosinophils, and, as a result, provide significantly different information from that obtained by the current standard of care.

Another approach, tethered capsule endomicroscopy (TCE)^18–22^, is a recently introduced endoscopy paradigm which allows for high-resolution, endomicroscopic imaging of the gastrointestinal tract in unsedated patients. Reports of tethered capsule endomicroscopy, demonstrated to date, have implemented optical coherence tomography (OCT) as the core imaging technology. These OCT-based TCE devices obtain cross-sectional architectural morphologic images (axial resolution ~10; lateral resolution ~30 µm) of the human esophagus. Studies suggest that OCT TCE can be useful for monitoring Barrett’s esophagus^19,23^. However, the resolution of OCT is insufficient to resolve individual eosinophils, which is required for the diagnosis of EoE.

Spectrally encoded confocal microscopy (SECM) is a higher resolution, high-speed, fiber optic-based reflectance confocal microscopy technique that, once incorporated in a capsule, could be well suited for cellular-level resolution imaging of the entire esophagus *in vivo*^24^. In the imaging console, SECM uses a rapid wavelength-swept source^24^ for tissue illumination and a single-element photodetector for detection of light reflected from the tissue. At the distal end of the SECM probe, light from an optical fiber is collimated via miniature optics onto a diffraction grating; spectrally dispersed light from the grating is focused into the tissue using a miniature objective lens. Light reflected from the sample returns through the lens, grating, and the core of the fiber. The fiber’s aperture serves as a spatial filter that rejects multiply scattered and out of focus light (as in conventional confocal microscopy). As the laser source wavelength is rapidly swept (λ_1_ → λ_2_), the focused beam at the distal end is scanned across a line in tissue, where each point along the line corresponds to a slightly different illumination wavelength. This unique configuration enables one to rapidly acquire one line of a confocal microscopy image without high speed moving parts and thus facilitates the miniaturization of confocal microscopy so that it can be incorporated into a small, flexible probe. A two-dimensional confocal image is formed by slowly rotating the SECM probe’s optics in a direction that is perpendicular to the spectrally encoded line. Kang *et al*.^25^ and Yoo *et al*.^26^ were first to use the SECM technology in a bench-top setting for interrogation of human esophageal biopsy samples. These *ex vivo* studies demonstrated the ability of SECM to resolve individual intraepithelial eosinophils as they were much more highly scattering than surrounding cells and SECM-visualized nuceli recapitulated the known bilobular morphology of eosinophil nuclei^27^. These *ex vivo* studies furthermore showed that there was a strong correlation between SECM and histology maximum eosinophil counts and that other leukocytes did not exhibit a similarly high reflectance confocal signal intensities^26^. In this paper, we report on technological and clinical feasibility of SECM TCE for comprehensive cellular-level imaging of the GI tract in unsedated human subjects.

## Methods

### SECM Tethered Endomicroscopy Capsule

#### SECM System

**Figure 1:**
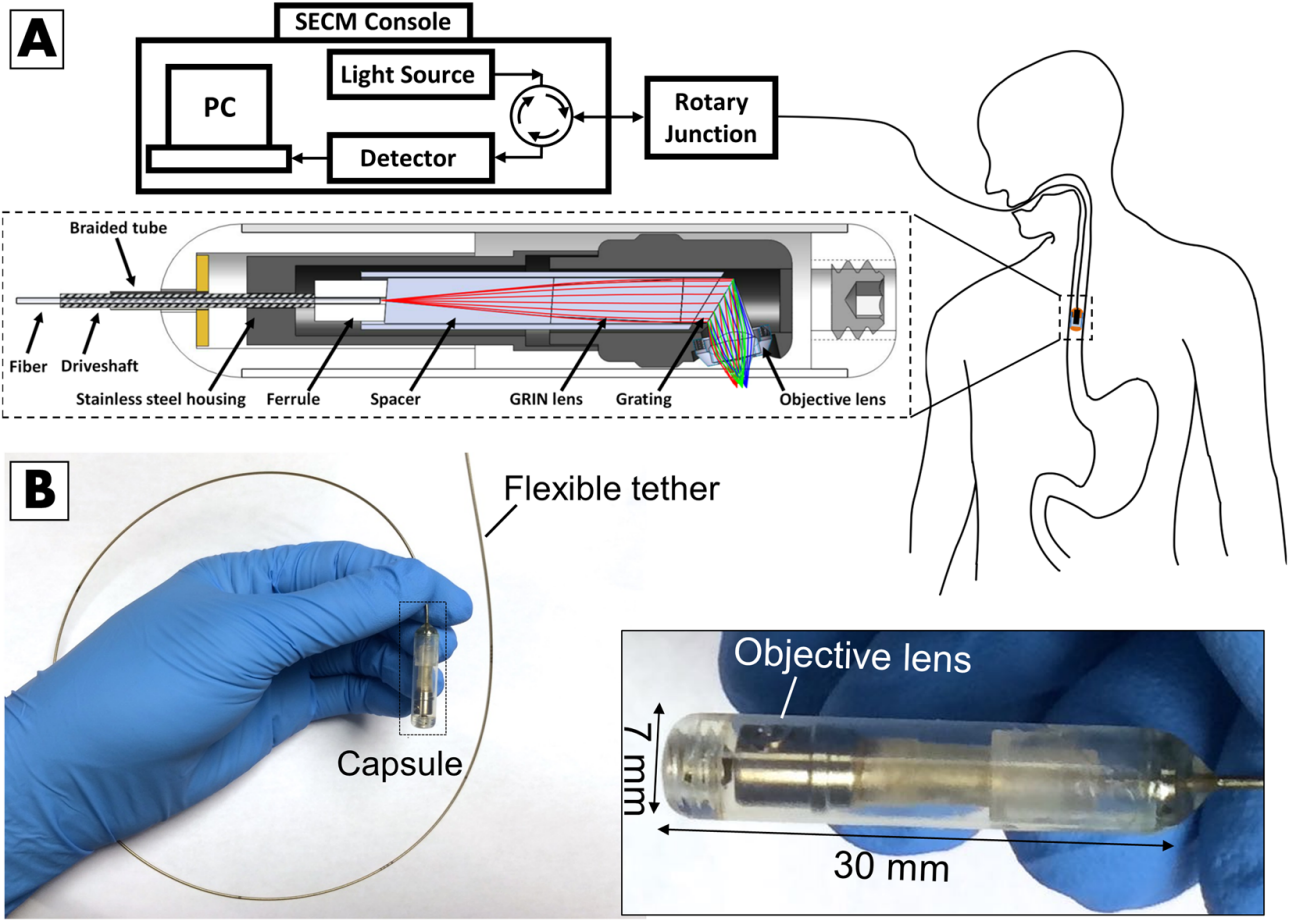
(**A**) Schematics of the SECM clinical system and tethered capsule. (**B**) Photograph of the clinical SECM capsule.

The SECM system used in this study was composed of three major sections, Fig. 1(A). The SECM console housed the imaging system, incorporating an InGaAs avalanche photodetector (Princeton Lightwave, USA) and an in-house built 100-KHz wavelength swept source (1,220 nm to 1,360 nm in 10 µs)^28^. Complete detail of the developed SECM imaging console can be found in prior publications^28,29^. The second major section of SECM system was the rotary junction. The rotary junction’s role was to maintain optical connection between the optical fiber emanating from the SECM console and the fiber within the tethered capsule while providing rotational torque to the inner components of the tethered capsule for spinning the internal optics at a speed of 6 revolutions/s^29^.

The third section was the tethered SECM capsule, Fig. 1(A), the development of which was previously described^29^. Briefly, the stainless steel housing was composed of two custom designed parts, manufactured through precision machining. The distal housing enabled positioning of the objective lens at a proper angle with respect to the diffraction grating^30^. The assembly process involved active alignment of the internal optical core assembly (i.e, fiber, ferrule, spacer, GRIN lens, diffraction grating assembly) with respect to the objective lens while iteratively measuring the axial resolution. Once the axial resolution was optimized, the internal parts were permanently fixed in the stainless steel housing using water resistant epoxy. The capsule’s body comprised a 7 mm (diameter) × 30 mm (length) transparent Fluorinated ethylene propylene (FEP) cylindrical shell enclosed by hemispherical acrylic caps. The shell enclosed miniature optics that redirected and focused wavelength swept light along a 200 µm-long spectrally encoded confocal microscopy line outside the capsule^30^. While a depth range of 100 µm was achieved by angling the spectrally encoded line as described in our previous publications^29,30^, we present the data as two-dimensional planes owing to the challenges of reconstructing three-dimensional data acquired in the dynamic, living patient enviroment. The capsule was connected to a flexible, 160-cm-long, 0.96 mm diameter sheath that served as a tether^29^. The sheath enclosed a driveshaft and an optical fiber; the fiber transmitted light to and received light from the miniature optics inside the capsule. The driveshaft conveyed rotational torque from the system’s optical rotary junction to the capsule’s optics to enable circumferential imaging. To achieve optimal imaging resolution, refractive index matching was employed in the capsule by filling it with sterile water. A photograph of the capsule is shown in Fig. 1(B). Lateral and axial resolutions of the SECM TCE capsule were 2.5 µm and 14 µm, respectively; the average distance of SECM focal line from capsule body was 100 µm; the area imaged in a single rotation of the capsule optics was 4.712 mm^2^ (23.562 mm [capsule circumference] × 200 µm [focal line length]); and the imaging speed was 6 revolutions/s.

#### Imaging Procedure

To examine the clinical feasibility of the tethered endomicroscopy capsule, n = 17 volunteers were enrolled for a first-in-human pilot study. After informed consent, unsedated subjects were asked to swallow the tethered SECM capsule while taking sips of water. With the operator loosely holding the tether, the capsule was gently allowed to descend through the esophagus to the stomach. The distance between the capsule and the incisors was recorded using marks on the tether that were spaced 5 cm apart. Images were visualized in real time to determine when the capsule had reached the stomach, evidenced by loss of contact and thus image data. Once in the stomach, the capsule was gradually pulled back up through the esophagus while imaging. A total of 4 imaging passes (4 up and 4 down) were performed in each subject. Immediately after the capsule was withdrawn, we asked each subject about pre-swallowing anxiety, whether the procedure was preferable to endoscopy, and the most difficult part of the procedure. Study data were collected and managed using REDCap electronic data capture tools hosted by Partners HealthCare Research Computing, Enterprise Research Infrastructure & Services (ERIS) group. REDCap (Research Electronic Data Capture) is a secure, web-based application designed to support data capture for research studies^31^. The study was reviewed and approved by the institution IRB (Partners IRB protocol #2013P000863) and performed according to all institutional and federal regulations and standards. The study was registered at clinicaltrial.gov on 11 June 2014 under number NCT02202590. According to regulations, each subject gave informed consent. Specific consent was obtained for use of images, audio, and video recording in medical research and education including in a professional journal or medical book or other media such as the Internet. While the consent form highlighted the fact that the face or voice of volunteers may be recognizable in published work, all subjects identity were removed by coding and visual features were blurred in the video.

#### Post-Procedure Image/Data Processing

After the procedures, the raw data was processed by custom software created in Matlab and Visual Studio platforms. This data processing step entailed background subtraction and data resampling to achieve identical aspect ratio in circumferential and longitudinal scanning directions. For this feasibility study, we did not cross-correlate consecutive SECM frames to correct for peristaltic movement and/or capsule slipping. As such, the SECM large images were simply constructed by stitching the acquired frames one after each other. We do not anticipate this stitching method to pose any diagnostic-related limitation for EoE patients as a single SECM confocal image strip entails an area corresponding to 24 conventional high-power fields (HPF), assuming a conventional microscopic HPF diameter of 500 µm. The HPF is a field of view that is used by pathologists to render a diagnosis of EoE where >15 eosinophils/HPF is considered positive^4^.

To study the imaging performance of the SECM TCE capsule procedures, representative pullbacks were selected for each subject. The travel length of capsule was then calculated by correlating the tether mark readings, which the operator reported every 5 cm, with SECM frame numbers. In addition, imaged lengths and areas were calculated for each representative pullback by multiplying the number of acquired confocal strips with a strip width (200 µm) and area (200 µm × 2.2 cm), respectively. Due to loss of contact between the capsule and stomach, not all of the imaged area was acquired from tissue. As such, the section of pullback distal to the gastroesophageal junction (GEJ) was first excluded and then the imaged tissue area was calculated via thresholding the gray level corresponding to tissue.

#### Capsule Reuse

After each imaging procedure, capsules were carefully examined under a microscope for any physical damage to ensure that the mechanical integrity of the capsule was not compromised for the next use. Then, capsules were connected to the SECM console for imaging of standard phantoms to ensure acceptable imaging performance of each capsule prior reuse. Finally, capsules were disinfected for reuse in accordance with the standard procedure for the disinfection of gastrointestinal endoscopes (i.e., submersion in Cidex OPA for 12 min).

#### Biopsies and Histology

Following SECM TCE, EoE patients underwent sedated standard of care endoscopy with biopsy, acquiring approximately 6 biopsies per case. Biopsies were embedded in paraffin, sectioned with a thickness of 5 μm, and subsequently stained with hematoxylin and eosin (H&E). All slides were digitized in their entirety using a whole slide scanner.

## Results

### Demographics, Procedure, and Subjects’ Feedback Data

A total of 17 subjects were enrolled for this study. The study cohort included healthy subjects (n = 4; 2 male and 2 female) and subjects previously diagnosed with EoE (n = 13; 12 male and 1 female). While 16 subjects went through the procedure without any complication, one EoE subject could not swallow the capsule. Since the post-procedure questionnaire was not completed by the EoE subject who was unable to swallow the capsule, the summary data and statistics provided below includes only demographic information and number of swallowing attempts for this subject.

The data extracted from post-procedure questionnaires revealed that 91.67% (11 out of 12) of EoE subjects, who completed the procedure, preferred the unsedated SECM capsule over endoscopy, while 8.33% (1 out of 12) had no preference between the two technologies. No EoE subject who completed the procedure preferred sedated EGD over the unsedated SECM capsule procedure. A significant majority of subjects (11 out of 17) identified the swallowing phase of the procedure as the most challenging; yet, 16 out of 17 (94.12%) managed to swallow the capsule without any complications. Four subjects found capsule removal to be the most challenging part. The mean procedure time for all 4 imaging passes through the esophagus (4 up and 4 down) was 11.63 ± 0.63 minutes. On average, a single pullback took about one minute. The average pre-swallowing anxiety level was found to be 1.69 (scale: 1 – not anxious at all; 5 – extremely anxious). A summary of subjects’ demographics, procedure data, and post-procedure feedback is presented in Table 1.

**Table 1:** Aggregated study data (average ± standard error of mean).

Population	Aggregated Data								
	*n*	Demographic Data			Procedure Data		Subject Feedback Data		
		Age	Race	Gender	# of Swallow Attempts	Total Capsule Procedure Time (minutes)	Pre-Swallowing Anxiety	Preference over EGD?	Hardest Part of Procedure
Presumed Normal Volunteers	4	31.75 ± 6.98	Caucasian/White	2 M; 2 F	1.25 ± 0.25	14 ± 1.08	1 ± 0.00	Yes 4; No 0; Not sure 0;	Swallow2; Tether 0; Removal 2; None 0
EoE Volunteers	13	23.08 ± 3.37		12 M; 1 F	1.92 ± 0.40	10.83 ± 0.63	1.92 ± 0.29	Yes 11; No 0; Not sure 1;	Swallow9; Tether 1; Removal 2; None 1
Total	17	25.12 ± 3.09		14 M; 3 F	1.76 ± 0.31	11.63 ± 0.63	1.69 ± 0.24	Yes 15; No 0; Not sure 1;	Swallow11; Tether 1; Removal 4; None 1

Pre-swallowing anxiety rubric: 1. not anxious at all; 2- a little anxious; 3 - moderately anxious; 4 - very anxious; 5 - extremely anxious.

### Imaging results

#### Summary data

Table 2 lists SECM TCE capsule imaging performance metrics for all subjects. The average length of esophagus that was imaged was 9.19 ± 2.25 cm. The average imaged area was 20.22 ± 4.94 cm^2^ however due to loss of contact the net tissue area imaged was found to be 13.52 ± 4.00 cm^2^ which is equivalent to ~7000 conventional microscopic HPF. The total image length was on average 52% of the total capsule travel length. The average raw data file size per capsule pullback was 30.03 ± 7.34 GB.

**Table 2:** SECM TCE Capsule imaging data for all subjects.

Subject #	Population	Capsule Travel Length (cm)	Imaged Length (cm)	Portion of Travel Length Imaged (%)	Imaged Area (cm^2^)	Tissue contact (%)	Imaged tissue (cm^2^)	Imaged tissue (HPF)	Data File Size of Pullback (GB)
1	Normal Volunteers*	15	3.88	25.83	8.53	65.58	5.59	2847	12.66
2		20	11.36	56.81	25.00	62.85	15.71	8002	37.12
3		24	12.51	52.11	27.51	71.35	19.63	9998	40.85
4		15	9.91	66.03	21.79	66.43	14.48	7373	32.36
5	EoE Volunteers	25	11.49	45.98	25.29	59.81	15.12	7703	37.55
6		27	12.25	45.37	26.95	80.93	21.81	11108	40.02
7		10	9.94	99.39	21.87	72.39	15.83	8062	32.47
8		Data not available; Subject could not swallow the capsule at 5 attempts.							
9		23	10.70	46.51	23.53	58.81	13.84	7048	34.94
10		17	8.38	49.28	18.43	67.59	12.46	6345	27.37
11		10	8.38	83.78	18.43	60.63	11.17	5691	27.37
12		21	8.07	38.43	17.76	66.74	11.85	6036	26.37
13		15	7.55	50.36	16.62	70.04	11.64	5928	24.68
14		20	6.93	34.65	15.25	51.73	7.89	4017	22.64
15		20	7.54	37.72	16.60	77.98	12.94	6591	24.64
16		17	9.69	56.99	21.32	73.51	15.67	7980	31.65
17		15	8.50	56.67	18.70	57.31	10.72	5458	27.77
All Volunteers (mean ± standard deviation)		18.38 ± 5.00	9.19 ± 2.25	52.87 ± 18.27	20.22 ± 4.94	66.48 ± 7.82	13.52 ± 4.00	6886.72 ± 2037.94	30.03 ± 7.34

The imaged length was computed as the number of acquired confocal image strips multiplied by the strip width (200 µm). Tissue contact was calculated from GEJ to the most proximal capsule imaging location in a single pull back. Imaged tissue was computed as imaged area multiplied by tissue contact. *Presumed to be normal.

Below, we present representative SECM TCE datasets that were obtained from this clinical study.

**Figure 2:**
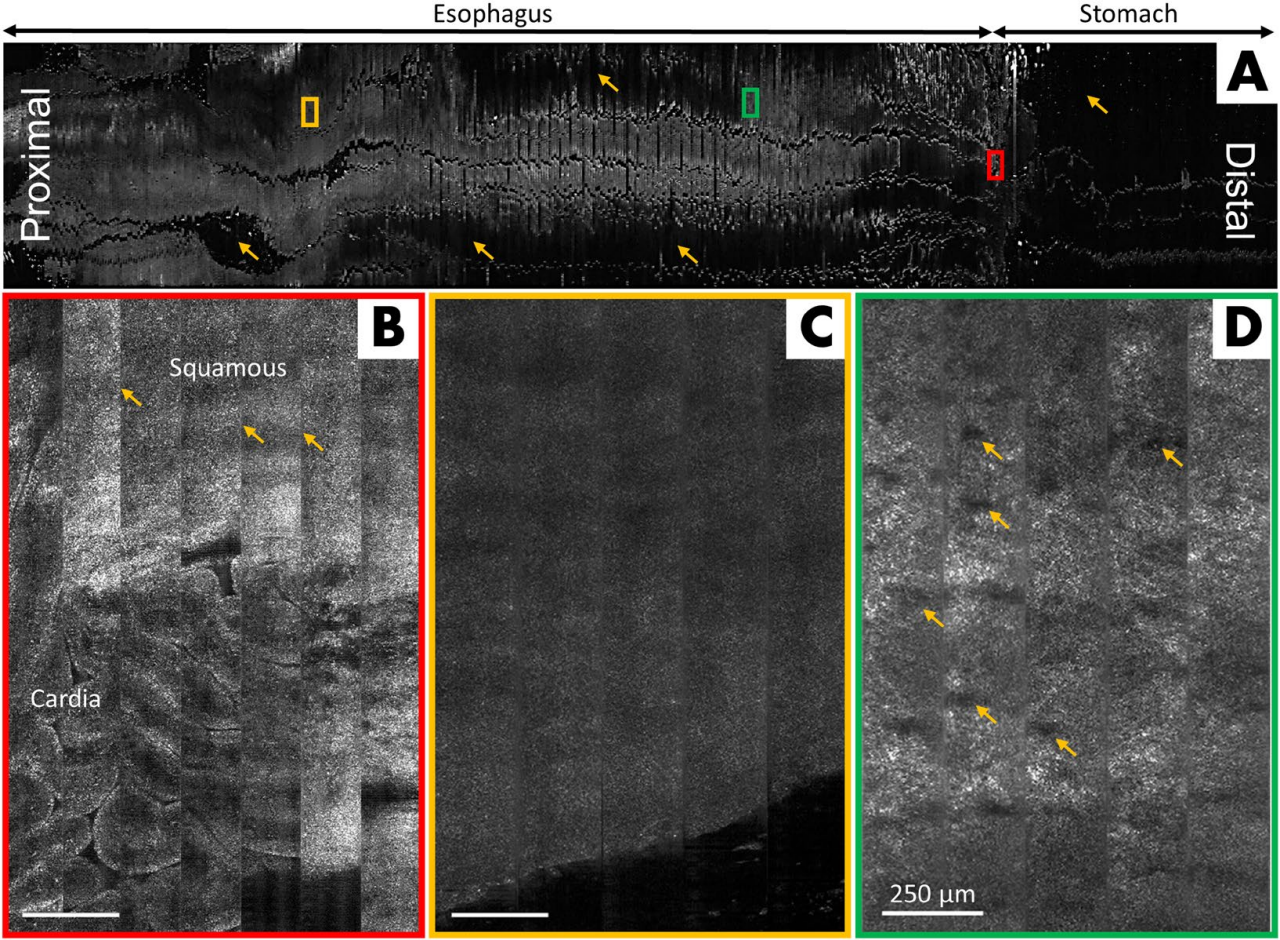
Representative SECM capsule images of normal esophagus, acquired *in vivo*. (**A**) Low-magnification view of the entire SECM image, spanning 2.2 cm × 15 cm. Arrows point to areas of loss of contact; (**B)** Magnified portion of (**A**, red box) showing the abrupt transition of gastric columnar epithelium to esophageal squamous epithelium at the gastroesophageal junction. Arrows point to the seam lines between adjacent confocal image strips; (**C**) Magnified region within (**A**, orange box) demonstrating a homogeneous appearance, representing ostensibly normal squamous epithelium; (**D**) Magnified view of region in (**A**, green box) showing lamina propria with papillae (arrows). Scale bars in **B**–**D** = 250 µm.

Figure 2 depicts a representative case of a presumably normal esophagus from a healthy volunteer. The low-magnification view of the entire confocal image, Fig. 2(A), is 2.2 cm × 15 cm (i.e., capsule circumference × pullback length). However, due to loss of contact (indicated by arrows in Fig. 2(A)), the net tissue area imaged was 14.48 cm^2^ which is equal to approximately 7,373 conventional microscopic HPFs.

Figure 2(B) depicts the characteristic SECM appearance of the gastroesophageal junction (GEJ) as seen by the capsule. The subimage is composed of confocal strips acquired during 7 consecutive rotations of capsule optics while the capsule was being pulled back by the operator. Existence of seam lines, arrows in Fig. 2(B), between the adjacent image strips is due to the fact that the TCE capsule incorporates a helical scanning scheme, rather than a raster scanning scheme used in *ex vivo* confocal microcopy. Consequently, the structural patterns do not precisely match at the interface of adjacent strips. Nevertheless, the transition from columnar epithelium of gastric cardia mucosa to squamous epithelium of esophageal mucosa is clearly captured by the SECM TCE device. Figures 2(C,D) show SECM capsule images of squamous epithelium and the lamina propria in the presumably healthy esophagus. Images of the epithelium are homogeneous owing to lack of contrast in cellular microstructures whereas papillae (arrows in Fig. 2(D)), can be visualized in the lamina propria. These observations are consistent with prior studies of the appearance of normal esophageal biopsy samples using SECM technology *ex vivo*^25,26^.

**Figure 3:**
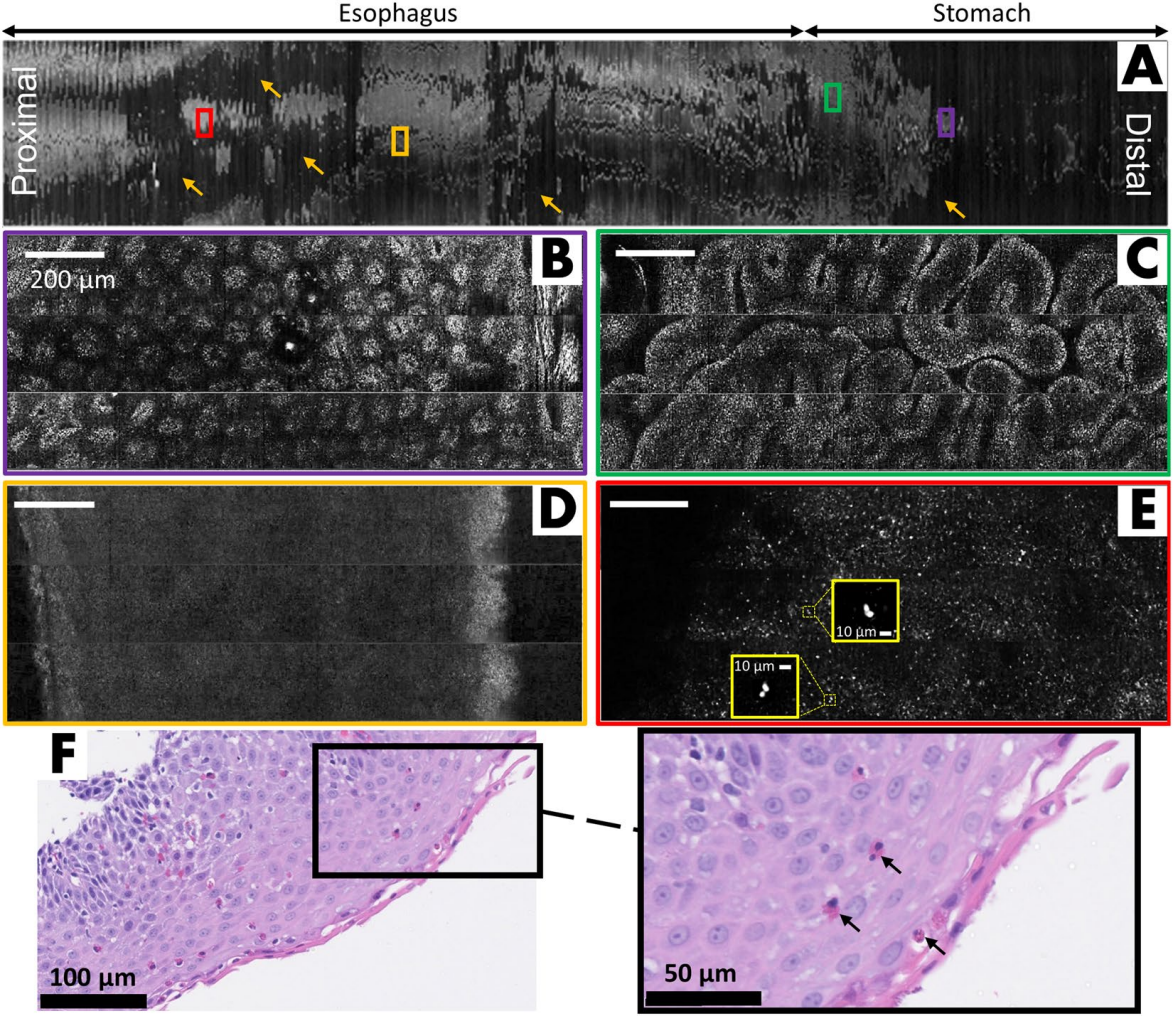
Representative SECM TCE images from an EoE subject, acquired *in vivo*. (**A**) Low-magnification view of the entire SECM image dataset, spanning 2.2 cm × 17 cm; (**B**) Magified portion of (**A**, purple box) demonstrating an oval crypt pattern consistent with body/fundic mucosa. (**C**) Magnified region of (**A**, green box) showing columnar epithelial morphology consistent with gastric cardia mucosa; (**D**) Magnified area from (**A**, yellow box) showing a characteristic homogeneous appearance suggestive of normal squamous epithelium; (**E**) High-magnification portion of a proximal region of (**A**, red box) showing an area of epithelium with irregularly distributed highly reflecting cells that are likely intraepithelial eosinophils. The characteristic bi-lobed structure of the eosinophil nucleus can be clearly resolved in many of these cells (yellow magified regions in (**E**)); (**F**) Representative histopathologic image of an esophageal biopy from the same subject confirming the presence of intraepithelial eosinophils (arrows). Scale bars in **B**–**E** = 200 µm.

Figure 3 depicts a case from a patient with a prior diagnosis of EoE. The low-magnification view of the entire confocal image, Fig. 3(A), is 2.2 cm × 17 cm. However, due to loss of contact, the net tissue area imaged was 15.12 cm^2^ which is equal to approximately 7,703 conventional microscopic HPFs. Magnified regions of the SECM dataset taken from the stomach (Fig. 3(B,C)) show oval crypt patterns of body/fundic mucosa and columnar epithelium that are typical of gastric cardia mucosa, respectively. Figure 3(D) is a high-magnification view of the homogeneous normal esophageal squamous epithelium. In contrast, another region in the SECM dataset (Fig. 3(E)) demonstrates an area of epithelium with irregularly distributed highly reflecting cells. In our previous benchtop SECM studies of biopsies from EoE patients^25,26^ such highly reflecting cells were confirmed histologically to be eosinophils. Particularly notable are the bi-lobed morphology of the eosinophil nuclei (Fig. 3(E)). Histopathologic examination of biopsies taken from this subject were in agreement with SECM capsule observations, demonstrating a similar distribution of esophageal eosinophils (Fig. 3F) and a positive diagnosis of EoE with a peak eosinophil count of >20/HPF.

**Figure 4:**
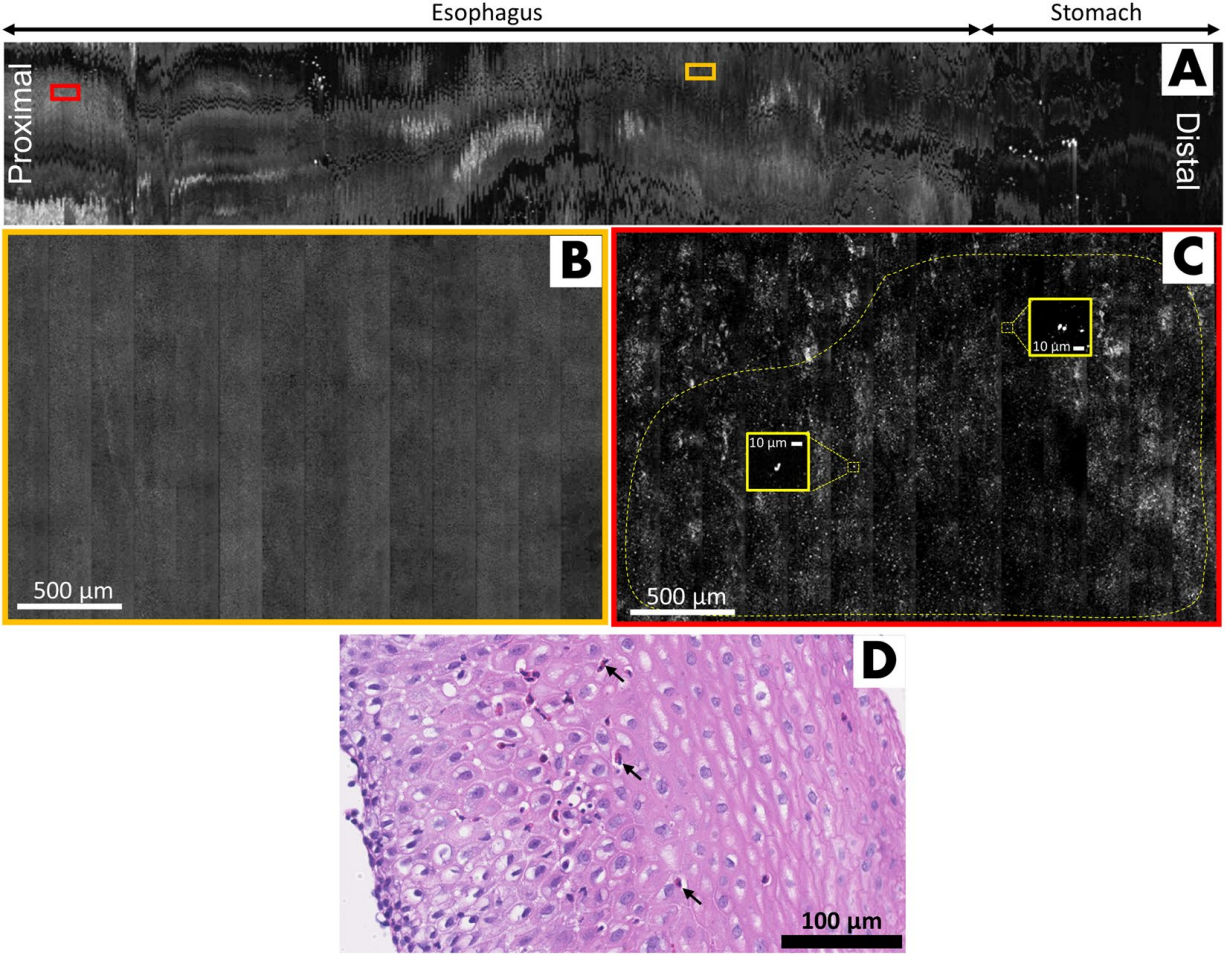
Representative SECM capsule images from an EoE subject, acquired *in vivo*. (**A**) Low-magnification view of the entire confocal image, spanning 2.2 cm × 25 cm; (**B**) Magnified portion of (**A**, orange box) showing homogeneous normal squamous epithelium; (**C**) Magnified region of (**A**, red box) demonstrating irregularly distributed highly reflecting cells consistent with intraepithelial eosinophils. The characteristic bi-lobed eosinophil nuclear morphology can be clearly seen (yellow insets); (**D**) Representative histopathologic image of an esophageal biopy from the same subject confirming the presence of intraepithelial eosinophils (arrows).

Figure 4 shows a representative case from another subject with a prior diagnosis of EoE. The low-magnification view of the entire confocal image, Fig. 4(A), is 2.2 cm × 25 cm, which approximates 19,600 conventional microscopic HPFs. Figure 4(B) is a high-magnification view of one region in the SECM image of the esophagus showing a uniform pattern that is suggestive of normal stratified squamous epithelium. In another portion of the SECM dataset, highly reflecting cells are seen (Fig. 4(C)), indicating the presence of intraepithelial eosinophils. As in the prior case, the characteristic bi-lobed appearance of eosinophil nuclei were resolved. Histopathology of biopsies taken from this subject were in agreement with the SECM capsule findings, demonstrating active esophagitis with a peak intraepithelial eosinophil count of 20 per HPF (Fig. 4(D)). Similar to the previous cases, other tissue features such as the gastric cardia and body/fundic mucosa were also clearly identified.

## Discussion

In this paper, we have presented first-in-human results from a pilot SECM TCE clinical study. To our knowledge, the confocal microscopy images acquired with this device represent the largest confocal images obtained *in vivo* to date. Our findings show that the device can successfully be utilized in unsedated subjects to acquire comprehensive, large area, cellular-resolution images of the esophagus. Procedure times were short (11.63 ± 0.63 minutes), patients tolerated the procedure well, and out of EoE subjects who completed the study, most (91.67%) preferred this exam over standard, sedated endoscopy. Comparison of values for average capsule travel length (18.38 ± 5.00 cm) and average imaged length (9.19 ± 2.25 cm) suggests that the pullback speed was not slow enough for the capsule to image the entire esophageal length. As a result of this undersampling, on average, 52.87 ± 18.27% of esophagus length was imaged in this pilot study. Assuming that a standard forceps biopsy spans 2 × 2 mm^2^, and considering that an average of 6 biopsies are collected from EoE patients in each EGD procedure, each pullback of the SECM TCE pilot study on average yielded about 56 times more imaged area for tissue diagnosis compared to sedated endoscopic biopsy procedures. Yet, the amount of imaged tissue area can be further increased by simply incorporating a slower pullback speed to avoid undersampling.

The ability of the SECM TCE capsule to more thoroughly image the esophagus is specifically important for diagnosis, monitoring, and management of diseases with a patchy nature such as EoE. Images of normal and EoE subjects clearly show the potential of this device to enable the diagnosis of eosinophilic esophagitis and possibly other esophageal disorders. While the results from this paper are preliminary, they do suggest that SECM TCE has the potential to become a promising diagnostic tool in gastroenterology.

Limitations of this study include a low number of patients, as it was a pilot study to demonstrate clinical feasibility of SECM TCE. Future work will include a prospective clinical study to determine the diagnostic accuracy of SECM. In addition, while representative biopsies and histology were obtained for EoE cases, we were unable to precisely co-register SECM image findings to histology, a shortcoming that can be overcome in the future by registering to landmarks and implementing laser marking technology that has been demonstrated in previously reported OCT esophageal probes^32^. While reflectance confocal images acquired by SECM show a great degree of detail, they do suffer from speckle noise, which is absent in fluorescence confocal microscopy datasets that are seen with CLE. Implementation of a double clad fiber in the TCE probe should overcome this issue^33^. In this study, the capsule descended or was pulled back too rapidly, resulting in the imaging length being smaller than the capsule’s travel length. This limitation can be mitigated by operator training or by implementing an automated tether pullback device. Another limitation we encountered involved the large size of the images. At an average of 30.03 ± 7.34 GB/Pullback, these datasets were difficult to manipulate and display. Software development, like that for whole slide imaging datasets is clearly merited to improve the ability to visualize all the data acquired by this technique. Further improvements should include development of machine and deep learning algorithms for automatic eosinophil counting to decrease the time required to review the datasets and render a diagnosis. Finally, resolution and contrast could be improved to extend this technology beyond counting eosinophils. Such advancements, including the use of higher NA lenses and the investigation of acetic acid to improve nuclear contrast^25,34^ are underway.
